# Randomized sham controlled trial of cranial microcurrent stimulation for symptoms of depression, anxiety, pain, fatigue and sleep disturbances in women receiving chemotherapy for early-stage breast cancer

**DOI:** 10.1186/s40064-015-1151-z

**Published:** 2015-07-23

**Authors:** Debra Lyon, Debra Kelly, Jeanne Walter, Harry Bear, Leroy Thacker, Ronald K Elswick

**Affiliations:** Kirbo Endowed Chair, University of Florida College of Nursing, Gainesville, FL 32601 USA; Virginia Commonwealth University School of Nursing, Richmond, Virginia USA; Massey Cancer Center, School of Medicine, Virginia Commonwealth University, Richmond, Virginia USA

## Abstract

**Purpose:**

Women with breast cancer may experience symptoms of depression, anxiety, pain, fatigue and sleep disturbances during chemotherapy. However, there are few modalities that address multiple, commonly occurring symptoms that may occur in individuals receiving cancer treatment. Cranial electrical stimulation (CES) is a treatment that is FDA cleared for depression, anxiety and insomnia. CES is applied via electrodes placed on the ear that deliver pulsed, low amplitude electrical current to the head.

**Methods:**

This phase III randomized, sham-controlled study aimed to examine the effects of cranial microcurrent stimulation on symptoms of depression, anxiety, pain, fatigue, and sleep disturbances in women receiving chemotherapy for early-stage breast cancer. Patients were randomly assigned to either an actual or sham device and used the device daily for 1 h. The study was registered at clinicaltrials.gov, NCT00902330.

**Results:**

The sample included N = 167 women with early-stage breast cancer. Symptom severity of depression, anxiety, and fatigue and sleep disturbances were generally mild to moderate. Levels of pain were low. Anxiety was highest prior to the initial chemotherapy and decreased over time. The primary outcome assessment (symptoms of depression, anxiety, fatigue, pain, sleep disturbances) revealed no statistically significant differences between the two groups, actual CES vs. sham.

**Conclusion:**

In this study, women receiving chemotherapy for breast cancer experienced multiple symptoms in the mild to moderate range. Although there is no evidence for the routine use of CES during the chemotherapy period for symptom management in women with breast cancer, further symptom management modalities should be evaluated to mitigate symptoms of depression, anxiety, fatigue, pain and sleep disturbances over the course of chemotherapy.

**Electronic supplementary material:**

The online version of this article (doi:10.1186/s40064-015-1151-z) contains supplementary material, which is available to authorized users.

## Background

The American Cancer Society in 2015 projected that in the United States, there will be 231,840 women diagnosed with breast cancer (Siegel et al. 2015). Most women will be diagnosed in the early stages of the disease (Stage I and II) and 90% of these individuals can expect to survive at least 5 years due to improvements in adjuvant chemotherapy and targeted hormonal therapies (Siegel et al. 2012). However, cancer treatments, and, perhaps the cancer itself, contribute to a number of distressing symptoms. In particular, the administration of systemic chemotherapy is associated with multiple, co-occurring distressing symptoms (Dodd et al. 2010; Goedendorp et al. 2008), which include depressive symptoms (Badger et al. 2007), anxiety (Badger et al. 2007), fatigue (Berger et al. 1997), and pain (Utne et al. 2010; Valeberg et al. 2008). In women with breast cancer, anxiety and sleep disturbances (Lee et al. 2004) are also common during the adjuvant chemotherapy treatment phase. These symptoms, both individually and collectively, are strongly associated with decreased quality of life. In addition to the distress related to these common symptoms, fatigue, depression, and physical complaints are associated with poor employment outcomes for breast cancer (Hansen et al. 2008).

Because symptoms tend to occur together and may have synergistic negative effects on quality of life, there is a movement towards examining “clusters” of co-occurring symptoms in persons with cancer and for testing interventions that might be effective for ameliorating more than one symptom (Dodd et al. 2001). Pain, depression, and fatigue have been identified as components of a notable cluster that may also include anxiety and sleep disturbances. Collectively, these symptoms can be described as “psychoneurological symptoms” (PNS) (Lyon et al. 2013). PNS may result in a significant decline in quality of life by contributing to adverse health outcomes over the active treatment period and into survivorship. To date, conventional therapies have not been effective for treating multiple concurrent symptoms. Given the limitations of conventional modalities for symptom management, many breast cancer patients report using a complementary or alternative medicine (CAM) modality in conjunction with conventional cancer treatment (Saghatchian et al. 2014). CAM is defined as “a group of diverse medical and health care systems, practices, and products that are not presently considered to be part of conventional medicine” (National Cancer Institute website). The most common reason given for using CAM modalities in individuals with cancer is the belief that CAM will assist with relieving pain and controlling side effects related to disease or treatment (Mansky and Wallerstedt 2006).

Cranial electrical microcurrent stimulation (CES) is a non-invasive modality that falls under the category described by the National Center for Complementary and Alternative Medicine (NCCAM) as “Veritable Energy Medicine.” The putative mechanism of CES is not completely understood; however, there are several interrelated theories of the mechanism of action of applied “energy” modalities. Both high and low intensity energy modalities are thought to initiate neuromodulation. High intensity energy modalities such as repetitive transcranial magnetic stimulation (rTMS) have now entered main stream psychiatric and neurologic practice. The mechanism for rTMS is thought to include the activation or inhibition of cortical activity depending on stimulation parameters. In contrast, the mechanism of action for low-intensity AC stimulation is less clear. CES is a form of alternating current (AC) stimulation that involves the application of current to infra- or supra-auricular structures (e.g., the ear lobes). Evidence from EEG suggests that CES leads to changes in alpha and beta frequency ranges, indicating potential neuroplastic and cognitive effects. Schroeder and Barr (2001) measured EEG activity during sham and AC stimulation and showed increases in low alpha (8–12 Hz) and high theta (3–8 Hz) activity (Schroeder and Barr 2001). A second hypothesis for the effects of CES effects of cranial AC stimulation is that CES has a primary effect on the peripheral nervous system that is secondarily transmitted to the central nervous system (Zaghi et al. 2009). CES may render its effects not by polarizing brain tissue, but rather via rhythmic stimulation that synchronizes and enhances the efficacy of endogenous neurophysiologic activity (Zaghi et al. 2009). Additionally, increases in blood and cerebrospinal fluid levels of specific neurotransmitters, including serotonin, norepinephrine, dopamine, and β-endorphin have been reported when CES was used for both 1 and 2 weeks (Shealy et al. 1998).

Although CES has been used as a modality for treating symptoms of depression, anxiety and insomnia in multiple studies over many years, results have not yet reached mainstream acceptance, possibly due to the lack of rigorously designed randomized clinical trials. There are many studies that have found positive effects of CES (Klawansky et al. 1995); however, most studies have not been methodologically rigorous. Most have been open trials, have had relatively small samples, and few have had designs that have included sham devices (Mindes et al. 2015). Yet, CES has several advantages over most current symptom management strategies and other CAM modalities that make its consideration worthwhile. As a self-administered, relatively low-cost, and portable modality, CES can be used in the home without disrupting daily routines already taxed by the demands of cancer treatments. Given the importance of examining innovative symptom management modalities in oncology practice for multiple, concurrent symptoms, the primary aim of this study was to examine the effects of CES on symptoms of depression, anxiety, pain, fatigue and sleep disturbances in women receiving chemotherapy for early-stage breast cancer.

## Methods

### Study design

The study was conducted in accordance with the Declaration of Helsinki and was approved by the Virginia Commonwealth University Institutional Review Board. Women with newly diagnosed stage I–IIIA breast cancer, scheduled to receive at least four cycles of adjuvant or neoadjuvant chemotherapy were referred by oncologists in the Massey Cancer Center and its affiliate sites from June 2009 through December 2012. Women were included in the sample if they had a diagnosis of stages I–IIIA breast cancer; a performance score <2 using the Eastern Cooperative Oncology Group criteria; and were scheduled to receive at least four cycles of adjuvant or neoadjuvant chemotherapy. Women were excluded from the study if they had: (1) previous chemotherapy; (2) dementia; (3) active psychosis; (4) history of seizure disorder; (5) any implanted electrical device, or (6) began or changed a medication regimen for depression or other psychiatric condition within 30 days prior to study enrollment.

After informed consent was obtained, participants’ medical records were abstracted for medical history and current medication use. The timing of the baseline data collection varied depending on patients’ start day of chemotherapy, but was always within 48 h of the initial chemotherapy. During the study, each participant was required to use the CES device daily for 1 h until 2 weeks after chemotherapy cessation. Symptom data were collected weekly. All participants completed a log to record CES use and the number of times that the device was used was assessed weekly. The time to complete all questionnaires at each time point was approximately 30 min. All data were collected either at the Massey Cancer Center and its affiliate sites (the initial, midpoint and post-chemotherapy data were collected in person) or via a weekly telephone call with study personnel.

### Intervention

The researchers conducted th is study using an Alpha-Stim cranial electrotherapy stimulator. Both the active and sham devices were provided on loan by Electromedical Products International, (2201 Garrett Morris Parkway, Mineral Wells, TX 76067-9034). The CES unit (Alpha-Stim^®^ 100 Microcurrent Stimulator) passes microcurrent levels of biphasic electrical stimulation via ear-lobe electrodes. The CES unit was preset to provide 1 h of 100 μA (sub-sensory level), modified square-wave biphasic stimulation on a 50% duty cycle at .5 Hz, and to automatically turn off at the end of 1 h. CES devices were pre-set at the factory to provide a maximum of 60 min of modified square-wave biphasic stimulation at 0.5 Hz and 100 μA, the lowest setting below the level of perception. Sham devices were identical; however, the electrodes did not transmit a current. Because the devices were pre-set at the factory, participants were unable to change the settings. The devices were dispensed by the investigation pharmacy. Participants were instructed to use the device for 1 h each daily.

### Power analysis

Data from our pilot, feasibility study (Lyon et al. 2010) was used to estimate mean values, correlations, and model-fitting mean square errors for power computations. Power calculations were computed using SASv9.1. The sample size for the proposed study was 166 participants, from which we assumed a 10% attrition rate, similar to the attrition rate in our preliminary study, yielding a final sample size of 150. Power analysis was performed for repeated measures assuming an overall a = 0.05 and a power of 90%. A Bonferroni correction was made to adjust for multiplicity resulting from five dependent variables, thus reducing each symptom alpha level to 0.05/5 = 0.01.

### Randomization

Participants were randomized using a computer-generated random number sequence with a block size of four. Randomization was stratified based on two treatment regimen groups: those who received chemotherapy every 2 weeks and those who received chemotherapy every 3 weeks. Treatment assignments were blinded to the study investigators and patients.

### Measures

In addition to a demographic questionnaire that included details about the stage and characteristics of the breast cancer diagnoses, comorbidities and life-style habits, several validated symptom measures were used to collect symptom data. The Hospital Anxiety and Depression Scale (HADS) is a 14-item self-report questionnaire developed to detect the presence and severity of both anxiety and depressive symptoms at the time of reporting. Participants rate (0–3) the severity of each symptom over a 7-day period. The possible score on the subscales is 0–21 for depression or anxiety and a possible total scale score of 0–42. The HADS has well-established reliability and validity for both depression and anxiety in women with breast cancer (Zigmond and Snaith 1983).

The brief pain inventory (BPI) short-form is a pain assessment tool that has well-established reliability and validity for adult patients with no cognitive impairment in trajectory studies of cancer and its symptoms. The BPI assesses the severity of pain, location of pain, pain medications, amount of pain relief in the past 24 h or the past week, and the impact of pain on daily functions. The “Usual pain” item was used to measure pain severity over time in this study (Cleeland and Ryan 1994).

The brief fatigue inventory (BFI) is a 9-item scale that taps into a single dimension of fatigue severity and the interference fatigue creates in daily life. The BFI is a clinically validated tool used to assess cancer-related fatigue and its impact on daily functioning. The “Usual fatigue” item was used to measure severity of fatigue over time (Mendoza et al. 1999).

The General Sleep Disturbance Scale (GSDS) is a 21-item scale that consists of 21 items that evaluate various aspects of sleep disturbance (quality and quantity of sleep, sleep onset latency, number of awakenings, excessive daytime sleepiness, and medication use) over the past week (Lee 1992). Items are rated on a scale ranging from 0 (never) to 7 (every day). The 21 items are summed to produce a total score with a possible range from 0 (no sleep disturbance) to 147 (extreme sleep disturbance).

Adverse events (AEs) were assessed over the phone at week weekly and in person at the mid-point chemotherapy data collection and at the final data collection two weeks after completion of chemotherapy by asking the participants the open-ended question: How are you feeling? AEs were reported by the investigator regardless of whether they were deemed to be related to the treatment. AEs were graded using the most current version of the National Cancer Institute Current Toxicity Criteria.

### Statistical analysis

Descriptive statistics were used to describe participants’ clinical and demographic characteristics, and the level of symptoms at each of the study time points. A longitudinal repeated measures model was used to compare the effects of the CES intervention group to the sham group over the chemotherapy treatment period until final data collection two weeks after the final chemotherapy. Data of all participants was entered into a secure study database. All participants with any post-baseline data were included in the final analyses on a per protocol basis. The primary endpoints of levels of depressive symptoms, anxiety, pain, fatigue and sleep disturbances were analyzed using a longitudinal repeated measures model which included one between subjects variable (Group: CES or Sham), one within subject factor (visit period: baseline period, treatment period, post-treatment period) and the interaction between group and visit. Participants with In addition to these factors, the model included covariates for age (years), menopausal status (pre- or post-menopausal) and body mass index (kg/m^2^). We tested a variety of variance–covariance structures including compound-symmetry, AR(1) and unstructured; the structure that fit the data and provided the most parsimonious model was used. The outcome variables for the baseline period were constructed from either 1 or 2 observations (number of observations M = 1.02, SD = 0.14) averaged for each subject, while the outcome variables for the treatment period were constructed from the mean of 2–11 (number of observations M = 5.91, SD = 1.62) observations per subject and the outcome variables for the post-treatment period were constructed from the mean of 1–22 (number of observations M = 9.99, SD = 4.53) observations per subject.

## Results

A total of 167 women were consented and enrolled to participate in this study. A flow diagram (Additional file 1: Fig. S1) details the progress of the prospective and actual participants through the trial.

### Sample characteristics

The mean age of participants was 51 ± 0.78 years. Table 1 describes demographic characteristics of participants and the differences between groups. Other clinical characteristics such as hormone status and treatments (type, timing, and duration of chemotherapy) are reported in Table 2. The majority of women (88.3%) had infiltrating ductal carcinoma and were diagnosed as having stage II (61.4%) breast cancer in accordance with criteria set by the American Joint Committee on Cancer and most participants (53.4%) had grade 3 tumors. The majority of participants were white (61.7%), married (57.7%), had greater than a high school education (82.0%), and were currently non-smokers (80.1%). Group equivalence differences in the active group and sham group for demographic characteristics and clinical factors were examined by conducting t-tests and Chi square analyses and there were no significant differences between groups.

**Table 1:** Demographic characteristics

	Sham (N = 81)	Active (N = 82)	Total (N = 163)	P-value
Age (mean ± standard error)	51.91 ± 0.97	51.04 ± 1.21	51.47 ± 0.78	0.57*
BMI (mean ± standard error)	30.61 ± 0.87	30.74 ± 0.99	30.68 ± 0.66	0.92*
Leisure Activity Score (mean ± standard error)	25.30 ± 2.99	22.44 ± 2.56	23.86 ± 1.96	0.47*
Life Style Scores (mean ± standard error)	2.82 ± 0.05	2.85 ± 0.04	2.84 ± 0.03	0.66*
Race (count (%))				
White	53 (65.43%)	48 (58.54%)	101 (61.96%)	0.37**
Other	28 (34.57%)	34 (41.46%)	62 (38.04%)	
Education (count (%))				
≤HS	17 (21.52%)	12 (14.63%)	29 (18.01%)	0.26**
>HS	62 (78.48%)	70 (85.37%)	132 (81.99%)	
Marital status (count (%))				
Currently married	46 (56.79%)	48 (58.54%)	94 (57.67%)	0.82**
Not currently married	35 (43.21%)	34 (41.46%)	69 (42.33%)	
Employment				
FT	44 (55.00%)	39 (47.56%)	83 (51.23%)	0.34**
Not FT	36 (45.00%)	43 (52.44%)	79 (48.77%)	
Menopausal status (count (%))				
Pre	30 (37.04%)	40 (48.78%)	70 (42.94%)	0.13**
Post	51 (62.96%)	42 (51.22%)	93 (57.06%)	
Smoking status (count (%))				
Yes	20 (24.69%)	11 (13.41%)	31 (19.02%)	0.07**
No	61 (75.31%)	71 (86.59%)	132 (80.98%)	
Alcohol use (count (%))				
Yes	41 (50.62%)	43 (52.44%)	84 (51.53%)	0.82**
No	40 (49.38%)	39 (47.56%)	79 (48.47%)	

* t-test.

** Chi-square test.

**Table 2:** Clinical characteristics

	Sham (N = 81)	Active (N = 82)	Total (N = 163)	P-value
Surgery (count (%))				
Mastectomy	25 (31.25%)	29 (35.80%)	54 (33.54%)	0.28*
Minimal surgery	29 (36.25%)	20 (24.69%)	49 (30.43%)	
Other	26 (32.50%)	32 (39.51%)	58 (36.02%)	
Histology (count (%))				
IDC	76 (93.83%)	68 (82.93%)	144 (88.34%)	0.03**
Other	5 (6.17%)	14 (17.07%)	19 (11.66%)	
Grade (count (%))				
1	5 (6.17%)	6 (7.32%)	11 (6.75%)	0.95*
2	32 (39.51%)	33 (40.24%)	65 (39.88%)	
3	44 (54.32%)	43 (52.44%)	87 (53.37%)	
Stage (count (%))				
I	21 (25.93%)	16 (19.51%)	37 (22.70%)	0.25*
IIA	31 (38.27%)	27 (32.93%)	58 (35.58%)	
IIB	15 (18.52%)	27 (32.93%)	42 (25.77%)	
IIIA	13 (16.05%)	12 (14.63%)	25 (15.34%)	
IIIB	1 (1.23%)	0 (0.00%)	1 (0.61%)	
Chemotherapy (count (%))				
AC	4 (4.94%)	7 (8.75%)	11 (6.83%)	0.49*
AC followed by Taxane	35 (43.21%)	29 (36.25%)	64 (39.75%)	
CMF	1 (1.23%)	0 (0.00%)	1 (0.62%)	
TC	31 (38.27%)	37 (46.25%)	68 (42.24%)	
Other	10 (12.35%)	7 (8.75%)	17 (10.56%)	
Neoadjuvant				
Yes	26 (32.00%)	23 (28.00%)	49 (30.10%)	0.50**
No	55 (68.00%)	59 (72.00%)	114 (69.90%)	
Duration of chemotherapy (weeks)	15.93 ± 5.37	16.47 ± 5.42	16.20 ± 5.39	0.54*
Lymphnodes (count (%))				
Yes	35 (44.30%)	44 (53.66%)	79 (49.07%)	0.24**
No	44 (55.70%)	38 (46.34%)	82 (50.93%)	
Hormonal therapy (count (%))				
Yes	40 (51.95%)	48 (59.26%)	88 (55.70%)	0.36**
No	37 (48.05%)	33 (40.74%)	70 (44.30%)	
Estrogen receptor (count (%))				
Yes	42 (51.85%)	48 (58.54%)	90 (55.21%)	0.39**
No	39 (48.15%)	34 (41.46%)	73 (44.79%)	
HER2 NeuExpression (count (%))				
Yes	15 (18.52%)	24 (29.27%)	39 (23.93%)	0.11**
No	66 (81.48%)	58 (70.73%)	124 (76.07%)	
Progesterone receptor (count (%))				
Yes	34 (41.98%)	33 (40.24%)	67 (41.10%)	0.82**
No	47 (58.02%)	49 (59.76%)	96 (58.90%)	
LongActingOpiods (count (%))				
Yes	16 (19.75%)	19 (23.17%)	35 (21.47%)	0.60**
No	65 (80.25%)	63 (76.83%)	128 (78.53%)	
NonSteroidal analgesics (count (%))				
Yes	20 (24.69%)	10 (12.20%)	30 (18.40%)	0.04**
No	61 (75.31%)	72 (87.80%)	133 (81.60%)	
Anxiolytic (count (%))				
Yes	24 (29.63%)	17 (20.73%)	41 (25.15%)	0.19**
No	57 (70.37%)	65 (79.27%)	122 (74.85%)	

* t-test.

** Chi-square test.

### Levels of psychoneurologic symptoms over time

Women in both groups reported having multiple concurrent symptoms across all time points, however, the level of symptoms were relatively low. Anxiety levels were highest at baseline and decreased over time while depressive and fatigue symptoms increased over time. Levels of pain and sleep disturbances fluctuated slightly across time; however they remained relatively stable throughout treatment and after chemotherapy cessation. Fatigue scores were lowest at baseline and trended upward overtime with highest scores at time point three after chemotherapy treatment had ended. Table 3 demonstrates the values of PNS over time for each group and differences between the CES and sham groups.

**Table 3:** Outcome measures by time point (Mean (SD))

Measure	Group	Time point 1	Time point 2	Time point 3
Anxiety	Total	7.34 (4.11)	4.69 (3.46)	4.29 (3.78)
	CES	7.09 (4.09)	4.40 (3.19)	4.07 (3.51)
	Sham	7.59 (4.13)	4.98 (3.72)	4.51 (4.04)
Depression	Total	3.04 (2.62)	4.13 (3.17)	4.55 (3.51)
	CES	3.03 (2.48)	4.24 (3.22)	4.47 (3.36)
	Sham	3.06 (2.78)	4.02 (3.14)	4.63 (3.67)
Fatigue	Total	2.29 (2.81)	3.14 (2.30)	3.33 (2.50)
	CES	1.95 (2.71)	3.15 (2.21)	3.34 (2.47)
	Sham	2.63 (2.89)	3.14 (2.39)	3.32 (2.55)
Pain	Total	1.35 (2.01)	1.17 (1.50)	1.23 (1.72)
	CES	1.24 (2.05)	1.25 (1.44)	1.14 (1.65)
	Sham	1.45 (1.98)	1.09 (1.56)	1.32 (1.80)
Sleep	Total	40.50 (24.28)	40.55 (20.08)	39.70 (20.66)
	CES	40.70 (24.83)	38.70 (18.28)	38.50 (20.19)
	Sham	40.30 (23.87)	42.44 (21.73)	40.91 (21.21)

### Group differences in primary outcome measures

There were no statistically significant group differences in levels of depression, anxiety, pain, fatigue and sleep disturbance (Table 4) at any of the study measurement points (pre-chemotherapy, mid-chemotherapy, and/or two weeks after completion of chemotherapy). There was also no time by group interaction.

**Table 4:** Analysis of primary outcome variables

Effect	Primary outcome variables														
	Anxiety			Depression			Fatigue			Pain			Sleep disturbance		
	df	F	p-value	df	F	p-value	df	F	p-value	df	F	p-value	df	F	p-value
Group	1,147	0.77	0.38	1,147	0.04	0.84	1,147	0.02	0.88	1,146	0.00	0.10	1,147	0.13	0.72
Visit	2,144	55.97	<0.01	2,145	17.11	<0.01	2,143	12.77	<0.01	2,143	1.14	0.34	2,143	0.06	0.94
G × V	2,144	0.01	0.99	2,145	0.61	0.55	2,143	1.50	0.23	2,143	2.55	0.08	2,143	0.98	0.38
Age	1,147	12.78	<0.01	1,147	4.26	0.04	1,147	15.34	0.01	1,147	3.46	0.07	1,147	18.36	<0.01
Menopausal status	1,147	2.68	0.10	1,147	2.58	0.11	1,147	16.06	<0.01	1,147	3.78	0.05	1,147	12.48	<0.01
BMI	1,147	0.05	0.82	1,147	0.09	0.76	1,147	0.58	0.46	1,147	3.87	0.05	1,147	0.50	0.48

### Adverse events

One participant in the actual CES group had a seizure on a day when she did not use the device. The AE was considered to be unrelated to her usage of the CES device. She was removed from the study after the AE occurred.

## Discussion

This double-blind, sham-controlled clinical trial did not detect any difference between the CES and sham CES for reducing psychoneurologic symptoms in women with early-stage breast cancer receiving chemotherapy. The low level of symptoms contrasts with prior studies such as a cohort study of 222 women with early breast cancer which found a point prevalence of depression, anxiety, or both (including borderline cases) was 33% at diagnosis (Burgess et al. 2005). In a study of 94 women with early-stage breast cancer that used Paroxetine for decreasing levels of depression and fatigue, 26 (28%) patients were significantly depressed at baseline, using a CES-D score of 19 or greater to indicate depression (Roscoe et al. 2005). Given that levels of symptoms were generally lower than anticipated it is possible that a floor effect mitigated potential benefits of CES in this sample. This contrasts with a double-blinded sham controlled trial of 115 participants with a primary diagnosis of an anxiety disorder, found significant positive effects of CES vs. sham for reducing symptoms of anxiety (*p* = 0.001, *d* = 0.94) and depression (*p* = 0.001, *d* = 0.78) from baseline to endpoint of the study (Barclay and Barclay 2014).

In this study of CES in women with breast cancer, we found that while most symptoms were in the mild range, the symptoms had different trajectories over time. Anxiety levels were highest at baseline, while depressive symptoms increased over time. These levels of similar to a recent longitudinal study of women with early-stage breast cancer that also found that pre-chemotherapy anxiety scores were significantly (p < 0.05) worse at baseline than cycle 4 day 1, whereas depression scores were significantly worse during treatment than at pre-chemotherapy (p < 0.05) (Sanford et al. 2010; Ancoli-Israel et al. 2014) newly diagnosed breast cancer found that depressive symptoms increased after 4 weeks of chemotherapy (Rissling et al. 2011). Levels of fatigue and pain were low at baseline and fatigue increased over time. This finding is consistent with other studies which have reported that fatigue increased in prevalence, severity, and disruptiveness after the start of treatment (Jacobsen et al. 1999). Levels of sleep disturbances were consistent over time, comparable to women in third semester of pregnancy (Lee and Gay 2004). This finding is similar to a recent studies of women with early stage breast cancer that found higher levels of sleep disturbances prior to chemotherapy, at mid-cycle and 6 months after initiating chemotherapy after but little variation in levels over time (Sanford et al. 2013).

The utility of CES was not supported in this study. A recent meta-analysis concluded that the effects of CES over sham did not demonstrate statistical significance in reported studies of patients with pain (SMD −0.24, 95% CI −0.48 to 0.01, P = 0.06). The authors concluded that due to this uncertainty, any clinical application of this modality would be most appropriate within a clinical research setting rather than in routine clinical care (O’Connell 2014). Although CES was not effective in this study for reducing symptoms, it is noteworthy that few trials have rigorously examined the use of any modality for reducing symptoms during the chemotherapy treatment period in cancer patients. Under-treatment of symptoms including depression persists in individuals with cancer. A recent study found that one-fourth of the patients had multiple clinically significant symptoms, whereas only 22.5% were free of any clinically significant symptoms (Reece et al. 2013). Despite the widespread use of CAM in persons with cancer, there have been few double-blinded randomized trials to examine the safety, feasibility and efficacy of CAM modalities.

### Limitations

Several limitations should be considered. In this trial, we used a “pragmatic” approach, enrolling women with early-stage breast cancer prior to chemotherapy, regardless of their current or anticipated risk for psychoneurologic symptoms (Thorpe et al. 2009). Although this approach led to the recruitment of a racially diverse sample of participants and a high acceptance rate for the study, it did not, by design, target participants with a higher risk for symptoms. Due to the low severity of symptoms, a possible floor effect may have evident, whereby symptoms did not reach the threshold for warranting intervention. In the future, setting a symptom threshold for inclusion criteria should be considered.

Secondly, we had participants with differences in the composition of chemotherapy, the number of cycles or components of the adjuvant medication such as anti-nausea or anxiolytic medications. In addition, the use of medications begun after the initiation of chemotherapy was not controlled. Thirdly, in a trial of this length, because CES was used from the initiation to the completion of chemotherapy, there were many different parameters of usage. Some participants used CES for as few as 6 weeks and some for as many as 32 weeks (depending on their type and schedule of chemotherapy). Although there was no statistically significant difference in number of weeks by group assignment, this wide variability is potentially problematic. In addition, the dose for CES was set at the lowest intensity (sub-sensory) so that the sham could be tested. It could be that the dose intensity was not adequate for this population. Alternatively, it could be that a “nocebo” response was induced by the lack of appreciable stimulation delivered by the devices, both actual and sham.

Several other potential methodological weaknesses are noted. The question used to monitor adverse events was general, and not specifically focused on side-effects related to CES. However, in the context of cancer treatment, general questions, potentially identifying serious adverse events is expected from researchers conducting clinical trials in patients undergoing active cancer treatment. Adherence to CES was also self-reported: future trials could consider a more robust technological method, such as a counter on the device, to measure adherence. In addition, we cannot exclude the possibility of a placebo effect in participants in both groups resulting from the contact with study personnel. Study personnel communicated with the study participants weekly: this communication could have been an “intervention” by itself.

## Conclusion

Further testing of CES in individuals with cancer who meet a higher cut-off for levels of symptoms may warrant consideration in the future. Although the level of symptoms in women in this sample of women with early stage breast cancer was relatively mild to moderate, the need for tailored symptom management strategies persists for cancer patients who are in different stages or treatment or who may have elevated psychoneurologic symptoms in the survivorship period. However, there is not yet evidence for the utility of CES for routine use in symptom management regimens for women with early-stage breast cancer during chemotherapy.

## Additional file

Additional file 1:
**Fig. S1.** Consort 2010 Flow Diagram.
