# Supplementary material for: Randomized sham controlled trial of cranial microcurrent stimulation for symptoms of depression, anxiety, pain, fatigue and sleep disturbances in women receiving chemotherapy for early-stage breast cancer
Source: Springerplus. 2015 Jul 23;4:369. doi: 10.1186/s40064-015-1151-z (PMC4584261; doi:10.1186/s40064-015-1151-z)
Supplement: Additional file 1: — Fig. S1. Consort 2010 Flow Diagram. [file 40064_2015_1151_MOESM1_ESM.docx]

**CONSORT 2010 Flow Diagram**

## Follow-Up

Lost to follow-up (give reasons) (**n=0**)

Discontinued intervention (give reasons) (**n=5**)

**4- Participants were too overwhelmed by treatment**

Analysed (**n=75**)
♦ Excluded from analysis (give reasons) (**n= 8**)

Did not complete protocol

## Analysis

Allocated to **Sham** (**n= 83**)

Received allocated intervention (**n= 83**)

Did not receive allocated intervention (give reasons) (n=3)

1. **Patient changed her mind before initiation of intervention**
2. **Patient enrolled in a different clinical trial that did not allow co-enrollment**

Lost to follow-up (give reasons) (**n=4**)

**Participant expired**

Discontinued intervention (give reasons) (**n=4**)

**3. Participants were too overwhelmed by treatment**

**1- Participant had a seizure (not related to trial)**

Analysed (**n=77** )
♦ Excluded from analysis (give reasons) (**n=7**)

Did not compete protocol

Allocated to **Active** (**n=84**)

Received allocated intervention (**n=84**)

Did not receive allocated intervention (give reasons) (**n=3**)

1. **Not eligible (had prior chemotherapy)**
2. **Patient changed her mind before initiation of intervention**

## Allocation

Randomized (n=167)

Excluded (n=235)

♦  Not meeting inclusion criteria (n=134)

♦  Declined to participate (n=41 )

♦  Other reasons (n=60)

Assessed for eligibility (n=**402**)

## Enrollment
